# TSH Variability of Patients Affected by Differentiated Thyroid Cancer Treated with Levothyroxine Liquid Solution or Tablet Form

**DOI:** 10.1155/2017/7053959

**Published:** 2017-05-09

**Authors:** Carlo Cappelli, Ilenia Pirola, Elena Gandossi, Claudio Casella, Davide Lombardi, Barbara Agosti, Fiorella Marini, Andrea Delbarba, Maurizio Castellano

**Affiliations:** ^1^Department of Medical and Surgical Sciences, Endocrine and Metabolic Unit, Clinica Medica, 2nd Medicina, Spedali Civili di Brescia, University of Brescia, 25123 Brescia, Italy; ^2^Department of Molecular and Translational Medicine, 3rd Division of General Surgery, Spedali Civili di Brescia, University of Brescia, 25123 Brescia, Italy; ^3^Department of Otorhinolaryngology, Spedali Civili di Brescia, 25123 Brescia, Italy

## Abstract

**Background:**

Recent guidelines from the American Thyroid Association (ATA) indicate that, in many patients affected by differentiated thyroid cancer (DTC), the serum TSH should be maintained between 0.1 and 0.5 mU/L. The purpose of this study was to evaluate the TSH variability of patients affected by DTC treated with liquid L-T4 formulation or in tablet form.

**Patients and Methods:**

Patients were eligible if (a) they were submitted to a total thyroidectomy and ^131^I remnant ablation for DTC in our institution and (b) they were classified low-risk patients according to ATA guidelines 2009. Patients were randomized (1 : 1) to receive treatment of hypothyroidism with liquid L-T4 or tablet form. The first check-up evaluation was made from 8 to 12 months after ^131^I remnant ablation. TSH values were established again after further 12 months.

**Results:**

A significant increase in TSH values (median) was observed in patients taking tablets [TSH (min–max): 0.28 (0.1–0.45) versus 0.34 (0.01–0.78) mIU/L, *p* = 0.041] as compared to those taking liquid formulation [TSH (min–max): 0.28 (0.1–0.47) versus 0.30 (0.1–0.55) mIU/L, *p* = 0.345].

**Conclusions:**

The use of L-T4 liquid formulation, as compared to that of tablets, resulted in a significantly higher number of DTC patients maintaining TSH values in range for the ATA risk score, reducing TSH variability over the time.

## 1. Introduction

Differentiated thyroid cancer (DTC), which includes both papillary thyroid cancer (PTC, 90%) and follicular thyroid cancer (FTC, 10%), is the most common form of thyroid cancer. Although the overall incidence of DTC is low (1/10,000), it appears to be increasing. While most patients diagnosed with DTC have a good prognosis, a significant proportion has persistent or recurrent disease. Management of DTC has undergone major changes in recent years—it is now known that DTC is not a homogeneous condition and rather than employ an aggressive approach for all patients, and the trend is to risk, stratify, and individualize the treatment according to the disease characteristics.

Surgery is the basis of initial management while thyroid-stimulating hormone- (TSH-) suppressive therapy is the cornerstone of follow-up therapy. TSH-suppressive therapy has been shown to be effective not only in those with persistent metastatic disease but also in “disease-free” patients to reduce the risk of recurrence. In addition to the body of evidence that TSH stimulates DTC growth both in vitro and in vivo, the results of a study by Pujol et al. showed that reduced TSH suppression is associated with an increased incidence of relapse and that a high level of TSH suppression (TSH ranging from 0.05 to 0.1 mU/L) is required for the effective management of DTC [1]. In another study from the National Thyroid Cancer Cooperative Study Group Registry in 1548 patients, TSH suppression improved overall survival in stage II patients with subnormal TSH versus normal or elevated serum TSH [2]. In higher stage patients (stages III and IV), overall survival and disease-specific survival were improved in patients who maintained subnormal to undetectable serum TSH levels. The importance of TSH suppression in general and the degree of suppression in particular in preventing recurrence and adverse clinical events have been taken into account in the recently updated guidelines from the American Thyroid Association (ATA) which indicate that in many patients, the serum TSH should be maintained between 0.1 and 0.5 mU/L, taking into account the initial ATA risk classification, thyroglobulin (Tg) level, its trend over the time, and the risk of TSH suppression [3]. However, the potential benefits of reaching the therapeutic goal must always be balanced against possible adverse effects of subclinical thyrotoxicosis including exacerbation of angina in patients with ischemic heart disease, increased risk for atrial fibrillation in older patients, and increased risk of osteoporosis in postmenopausal women.

Individuals have a unique thyroid function, and although individual thyroid concentrations are maintained within relatively narrow limits, Andersen et al. reported large variations among individuals and thus a low index of individuality for thyroid function tests [4, 5]. They concluded that conventional population-based reference intervals for thyroid function tests may not be able to detect abnormal test results that are outside the normal range for an individual being tested.

While TSH suppression following surgery is an effective therapeutic strategy for the management of DTC, the importance of maintaining a constant level of suppression should not be underestimated for a number of important reasons including avoiding transient supraphysiological hormone concentrations; ensuring that variations in individual TSH levels are maintained within relatively narrow limits to correspond with the normal physical situation, TSH suppression is adequate and constant to ensure both suppression of growth and prevention of occurrence.

Levothyroxine (L-T4) is used worldwide as a replacement therapy for patients with hypothyroidism and in athyreotic patients following thyroidectomy for thyroid cancer [6], representing the third most common medication dispensed in the United States over the last few years [3]. Although replacement therapy with L-T4 has been prescribed for more than 60 years and is generally considered straightforward, cross-sectional surveys of patients undergoing treatment with levothyroxine demonstrate that between 40% and 48% are either overtreated or undertreated [7, 8]. In recent years, a number of novel formations of L-T4 including soft-gel capsules and liquid preparations have been marketed. The efficacy of the liquid L-T4 formulation, as compared with tablets, resulted in a significant reduction in TSH variability in hypothyroid patients, both in young people and in the elderly [9–11].

In one study to compare the effectiveness of L-T4 liquid formulation, with L-T4 tablets, in 152 hypothyroid patients without malabsorption or drug interference, patients were switched from the L-T4 therapy in tablets to liquid L-T4 at the same dosage, 30 min before breakfast. Serum thyrotropic hormone (TSH), free thyroxine (FT4), and free triiodothyronine (FT3) were re-evaluated after 1–3 months (first control) and 5–7 months (second control) from the switch. TSH values significantly declined with respect to the basal value after the switch to liquid L-T4 both at the first control (*p* < 0.05) and at the second control (*p* < 0.01); FT4 and FT3 levels were not significantly changed. We show that liquid L-T4 is more effective than L-T4 tablet in controlling TSH levels in hypothyroid patients without malabsorption, gastric disorders, or drug interference [12].

The aim of the present study was to evaluate the TSH variability in patients with DTC treated with the liquid and tablet formulations of L-T4.

## 2. Subjects and Methods

In the present study, patients affected and treated by differentiated thyroid cancer were randomly assigned to receive in the morning levothyroxine in tablets or in liquid solution.

Patients were recruited by those treated and followed at the Thyroid Unit of the Department of Clinical and Experimental Sciences, University of Brescia, Italy.

Patients' enrollment took place from January 2012 to February 2015. They were eligible if (a) they were submitted to a total thyroidectomy and ^131^I remnant ablation for differentiated thyroid cancer in our institution and (b) they were classified as low-risk patients according to ATA guidelines 2009 [13].

After assessing the eligibility, patients were randomized (1 : 1) to receive treatment of hypothyroidism with liquid L-T4 (Ibsa Farmaceutici Italia srl, Lodi, Italy) ingested during each patient's normal breakfast or tablets (Merck Serono S.p.A., Italy) ingested before breakfast after fasting overnight and 60 minutes prior to food ingestion.

In order to avoid any possible confounding variables, patients taking any medications known to interfere with L-T4 absorption [14] were excluded from the study. The intake of drugs potentially interfering with L-T4 absorption (in particular iron or calcium supplements and proton pump inhibitors) was monitored and recorded during the study period. Adherence to protocol requirements was assessed by a physician via personal interviews.

The first check-up evaluation was made from 8 to 12 months after ^131^I remnant ablation in all the patients, who were also assessed for thyroid-stimulating hormone. Serum concentrations of TSH were established again after a further 12 months. All participants had to maintain the same dosage of L-T4 therapy for the entire period of the study.

The study was approved by an independent institute review board and conducted in accordance with the Declaration of Helsinki and the Good Clinical Practice Guidelines of the International Conference on Harmonisation. All the participants provided prior written informed consent.

Serum concentrations of TSH (normal range: 0.2–4.2 mIU/L, analytical sensitivity 0.004 mIU/L; intra- and interassay coefficient of variation, 2.5 and 5.7%, resp.) and of FT4 (normal range: 8.0–18 pg/mL, analytical sensitivity 1 pg/mL; intra- and interassay coefficient of variation, 2.4 and 6.8%, resp.) were measured using a fully automated Architect i2000 analyser (Abbott Diagnostics, Abbott Park, IL, USA) based on chemiluminescent magnetic immunoassay.

## 3. Statistical Analysis

Statistical analyses were performed using SPSS 20.0 software (SPSS Inc., Evanston, IL, USA). Data are presented as mean ± standard deviation for parameters with normal distribution (age, weight, etc.). Normal distribution was checked using the Shapiro-Wilk test. TSH distribution level results were nonnormally distributed and were not normalised by the usual procedures of data transformation; in these cases, the results are presented as a median, with minimum and maximum values. Comparisons between continuous variables were performed by paired sample *t*-tests or by the Wilcoxon signed rank test of related samples, as appropriate. Two-tailed *p* < 0.05 was considered statistically significant.

## 4. Results

From January 2012 to February 2015, 102 patients (80 females and 22 males, aged 57.7 ± 11.2 years) assessed and treated by DTC were eligible for the inclusion criteria and were enrolled for the study. All the patients were submitted to total thyroidectomy and ^131^I remnant ablation for papillary thyroid carcinoma within three months from surgery at least. The day after radiometabolic treatment, all the patients started replacement therapy with levothyroxine at the dosage of 1.9 mcg/kg/day. The half of patients were in replacement therapy with liquid L-T4 ingested during each patient's normal breakfast, and the other 51 were on tablets ingested before breakfast, 60 minutes prior to food ingestion. Patients on tablets and undergoing liquid L-T4 therapy had been on the respective treatment since they began iodine remnant ablation and were never switched to another treatment.

The first check-up evaluation was made from 8 to 12 months after ^131^I remnant ablation in all the patients: the demographics and clinical characteristics are shown in Table 1. There were no significant differences in age, gender, weight, tumour size, L-T4 dosage, and TSH serum levels between patients on levothyroxine liquid solution and those taking it in tablet form.

**Figure 1:**
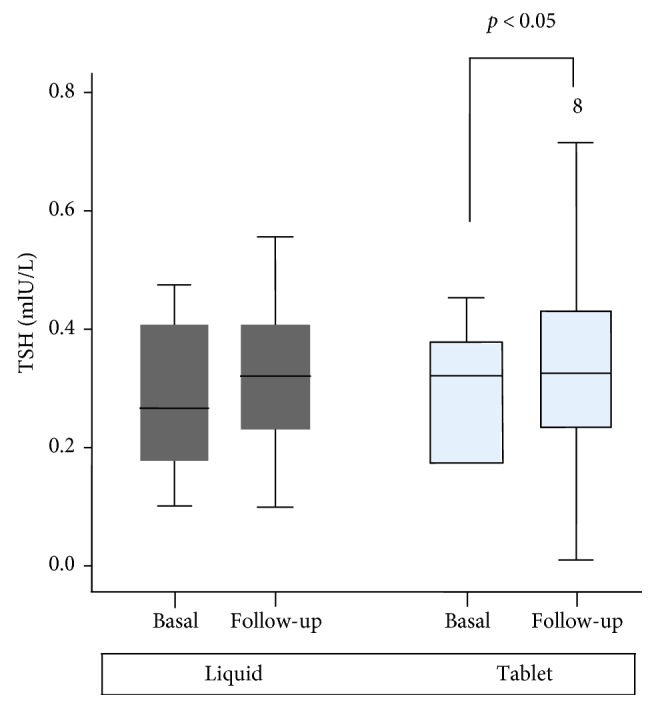
TSH values at first check-up (i.e., 8–12 months after ^131^I remnant ablation) and at follow-up (i.e., after 12 months from first check-up) in patients taking tablets and/or liquid L-T4 formulation.

Serum concentrations of TSH were established again after a further 12 months during the annual check-up (Figure 1). A significant increase in TSH values (median) was observed in patients taking tablets [TSH (min–max): 0.28 (0.1–0.45) versus 0.34 (0.01–0.78) mIU/L, *p* = 0.041] as compared to those taking liquid formulation [TSH (min–max): 0.28 (0.1–0.47) versus 0.30 (0.1–0.55) mIU/L, *p* = 0.345]. In detail, 7/51 (14.2%) subjects on tablets and 2/51 (3.9%) on liquid formulation showed TSH levels greater than 0.5 mIU/L; on the other hand, one patient on tablets and none in liquid treatment showed TSH values under 0.1 mIU/L. Body weight (kg) did not change from recruitment to check-up both for patients ingesting tablets (61.8 ± 9.2 versus 60.7 ± 9.4, *p* = 0.56) and for those on liquid L-T4 formulation (59.5 ± 12.8 versus 59.4 ± 12.7, *p* = 0.968).

## 5. Discussion

The present study comparing the tablet and liquid formulations of L-T4 in patients affected by differentiated thyroid cancer showed that liquid formulation resulted in reduced TSH variability over the time.

While recent years have seen a marked increase in new diagnoses of differentiated thyroid cancer, the number of thyroid cancer-related deaths has not changed over the time. This may be explained not only by improvements in education and socioeconomic status [15], easier access to diagnostic evaluations that enable the diagnosis of smaller cancers [16], but also by the multidisciplinary approach to thyroid cancer which has proved effective in reducing disease progression and death [17]. Moreover, adherence to check-up examinations [18] and the optimisation of TSH-suppressive therapy with L-T4 contribute to achieving this objective and to improving quality of life [19, 20].

In accordance with the ATA guidelines of 2009, low-risk patients should be treated with L-T4 to maintain TSH levels between 0.1 and 0.5 mIU/L [13]. This concept was extended in the most recent ATA guidelines in patients with an incomplete biochemical response to therapy, taking into account the initial ATA risk classification, thyroglobulin (Tg) level, its trend over the time, and the risk of TSH suppression [3]. Hesselink et al. have, in fact, shown that, in patients affected by DTC, a lower TSH level is associated with increased cardiovascular mortality, independent of age, sex, and cardiovascular risk factors, more than the DTC itself [21]. Furthermore, it is known that there is an increased risk of atrial fibrillation and osteoporosis with lowering serum TSH over time independently of the levels of FT3 and FT4 [21–24]. This finding indicates the importance of TSH variability during L-T4 treatment, in particular in patients with a small therapeutic window.

The recent introduction of nontablet formulations of L-T4 in the therapeutic environment seems to call this important issue into question. Negro et al. reported interesting data in this respect, showing that the administration of a liquid L-T4 formulation compared to tablets resulted in a significantly higher number of hypothyroid patients who remained euthyroid at the 12-month check-up, with a significant reduction of variability in TSH values [10]. We have also observed, in a retrospective series of 369 elderly hypothyroid patients, a greater stability in the thyroid profile of subjects treated with liquid thyroxine than of those treated with tablet formulation at the five-year check-up [11]. The most convincing explanation of this difference of stability is that the absorption of the liquid formulation is not affected by changes in gastric pH, as is the case for the tablets. Centanni et al. have in fact demonstrated that patients with impaired acid secretion require an increased dose of thyroxine, confirming that normal acid gastric secretion is necessary for the effective absorption of oral L-T4 [25]. Many recent reports have clearly demonstrated that liquid L-T4 formulation circumvents this problem in patients taking proton pump inhibitors, those suffering from gastric-related T4 malabsorption, those ingesting T4 with coffee or fed by enteral tube, and also those submitted to bariatric surgery [26–32]. Recently, Giusti et al. showed that, in patients affected by DTC, the risk of inadequate TSH levels seems to be more reduced in patients on liquid L-T4 than in those on tablets [17]. This study confirms and extends the previous one in a longer period of follow-up. We, in fact, demonstrated a significant reduction in TSH variability among patients assuming liquid L-T4 as compared to those on tablets. As a matter of fact, serum TSH values were out of range for the ATA guidelines (0.1–0.5 mlU/mL) more frequently in patients ingesting tablets than in those on liquid L-T4 formulation at the one-year follow-up [8/51 (15.7%) versus 2/51 (3.9%); *p* < 0.05, OR 4.9, 95%CI 1.0–24.7]. It is important to underline that no changes in body weight were observed among our patients, both for those ingesting tablets (*p* = 0.569) and for those on liquid L-T4 formulation (*p* = 0.968) from recruitment. This is of particular interest, because of the average replacement dose of L-T4 is calculated on body weight per day (approximately 1.6–2.0 mcg/kg/day), changing with the increase or decrease in the body weight itself [33].

The liquid formulation is currently only available in Italy. This is a possible limitation of the present survey, since all the clinical studies have been conducted in this country, among people belonging to the same ethnic group; accordingly, further studies performed in other countries are needed.

In conclusion, the use of L-T4 liquid formulation, as compared to tablets, resulted in a significantly higher number of DTC patients maintaining TSH values in range for the ATA risk score, reducing, at the same time, variability in TSH values.

## Figures and Tables

**Table 1:** Baseline demographics and clinical characteristics of the patients at the first check-up.

	Patients on liquid L-T4 (51 patients)	Patients on tablet L-T4 (51 patients)	*p* value
Age (yrs)	58.1 ± 11.6	56.9 ± 11.9	0.607
Gender (M/F)	9/42	13/38	0.327
Mean tumour size (cm)	1.6 ± 0.9	1.7 ± 1.0	0.596
Weight (kg)	59.5 ± 12.8	61.8 ± 9.2	0.303
L-T4 dosage (mcg)	128.7 ± 20.4	132.1 ± 20.4	0.407
TSH (mIU/L)	0.2 (0.1–0.4)^∗^	0.2 (0.1–0.4)^∗^	0.939
FT4 (pg/mL)	14.3 (10.9–17.4)	14.2 (10.7–17.7)	0.80

∗ indicates median (min–max).
